# HIV and neoplasms: What do we know so far?

**DOI:** 10.31744/einstein_journal/2023RW0231

**Published:** 2023-06-06

**Authors:** Thais Faria de Souza, Yasmin Vianna Sym, Ethel Zimberg Chehter

**Affiliations:** 1 Centro Universitário FMABC Santo André SP Brazil Centro Universitário FMABC, Santo André, SP, Brazil.

**Keywords:** HIV, Neoplasms, Acquired immunodeficiency syndrome, Antiretroviral therapy, highly active, Carcinogenesis

## Abstract

**Introduction:**

The human immunodeficiency virus (HIV) pandemic remains an important issue. In 2020, approximately 37.7 million people were living with the disease and there were more than 680 thousand deaths due to complications linked to the disease. Despite these exorbitant numbers, the introduction of highly active antiretroviral therapy has marked a new era, changing the epidemiological profile of the infection and related pathologies, including neoplasms.

**Objective:**

We performed a literature review to assess the role of neoplasms in patients with HIV after the introduction of antiretroviral therapy.

**Methods:**

A literature review was conducted based on the Preferred Reporting Items for Systematic Reviews and Meta-Analyses (PRISMA) method, searching the MEDLINE, LILACS, and COCHRANE databases for articles published from 2010 onwards.

**Results:**

Using specific key terms, 1,341 articles were identified; two were duplicates, 107 were selected for full-text evaluation, and 20 were included in the meta-analysis. The selected studies included 2,605,869 patients. Fifteen of the 20 articles indicated a reduction in the global incidence of AIDS-defining neoplasms and 12 indicated an overall increase in non-AIDS-defining cancers after the introduction of antiretrovirals. This growth trend could be explained by a range of factors including the aging population with HIV, risky behaviors, and coinfection with oncogenic viruses.

**Conclusions:**

There was a decreasing trend in the incidence of AIDS-defining neoplasms and increasing trend in non-AIDS-defining neoplasms. However, the carcinogenic effect of antiretrovirals could not be confirmed. In addition, studies focusing on the oncogenic role of HIV and screening for neoplasms in individuals with HIV are required.

## INTRODUCTION

Given the high prevalence and marked mortality associated with HIV, current discussions on this topic are highly relevant. According to statistical data collected by the Joint United Nations Program on HIV/AIDS (UNAIDS), approximately 37.7 million people were living with HIV (PLHIV) worldwide by the end of 2020. Among them, 84% were aware of their serological status, and 73% had access to antiretroviral therapy. Since the beginning of the global epidemic, approximately 79.3 million people have been infected with HIV and approximately 36.3 million people have died from AIDS-related illnesses. With regard to new HIV infections in 2020 (1.5 million), a 52% reduction has been noted since its peak in 1997.^(1)^ Furthermore, according to UNAIDS data from 2021, the risk of death from COVID-19 infection among individuals infected with HIV is twice than that of the general population.

The disease caused by HIV is characterized by a progressive decline in CD4^+^T lymphocytes leading to immunodeficiency. Its pathogenesis involves the life cycle of the virus and the host’s immune response. As the disease progresses in the absence of virus containment strategies, CD4^+^T cells are depleted steadily within lymphoid tissues, resulting in an increase in the percentage of viruses found in the plasma.^(2)^ This severe impairment of the immune system increases the risk of opportunistic infections and neoplasms related to immunodeficiency. Consequently, this worsens the outcome of the infection.^(3)^ Therefore, a lack of adequate treatment triggers a progressive immunodeficient state.

Since 1984, several therapies have been developed to manage the disease in people with HIV infections. In 1996, with the introduction of protease inhibitors (PIs), an era of highly active antiretroviral rherapy began. Notably, improvements in clinical outcomes, quality of life, and life expectancy were achieved. Furthermore, a marked decrease in patient morbidity and mortality was noted. The aim of antiretroviral therapy is to decrease HIV replication and reduce the risk of the immune system to be compromised, hence, reducing the risk to acquire AIDS. This has contributed to a notable restoration of the immune system, leading to a decrease in adverse events and isk of viral mutations, thereby improving the quality of life and maintaining and recovering the health of patients.^(2)^

The relationship between cancer and HIV/AIDS has been known since the beginning of the pandemic. The risk of neoplasms is higher in infected individuals than in the general population. AIDS-defining neoplasms occur with higher recurrence in these patients. For example, Kaposi’s sarcoma (KS), non-Hodgkin’s lymphoma (NHL), and invasive cervical carcinoma of the uterine cervix are 3,640, 77, and 60 times more common in PLHIV than in the rest of the population, respectively.^(4)^ Moreover, they occur more frequently in patients with advanced immunosuppression, and their prevalence was higher in the pre-antiretroviral therapy era.^(4)^ Such neoplasms are usually associated with co-infection with oncogenic viruses such as HHV-8 in KS, Epstein-Barr Virus (EBV) in NHL, and HPV in cervical squamous cell carcinoma.^(5)^

Malignant diseases that are not directly correlated with HIV infection are referred to as non-AIDS-defining neoplasms. In relation to these, a survey conducted in the USA and Puerto Rico in 1998 found a 37, 7.6, 4.5, 3.5, and 2.9 times higher risk of developing angiosarcoma, Hodgkin’s disease, multiple myeloma, brain cancer, and seminoma, respectively, in PLHIV compared to the risk of the general population.^(6)^

The link between cancer and HIV has evolved since the implementation of new treatments for AIDS.^(7)^ Although the relationship between HIV infection and neoplasms has been studied since the beginning of the pandemic, there is still a lack of information on this subject in the literature.

## JUSTIFICATION

With the advent of antiretroviral therapy, there has been an improvement in the immunological status of patients with HIV infections, and a reduction in the risk of progression to AIDS, resulting in a prolonged PLHIV life. Investigation of changes in the incidence of AIDS-defining and non-AIDS-defining neoplasms in patients treated with antiretroviral therapy is essential to understand their causes and provide cues to manage them. There is a need for studies on the reduced morbidity and mortality of PLHIV in the antiretroviral therapy era to understand how the therapy functions; whether it is due to the improved immunity of these patients, increased survival, the effects of antiretroviral therapy itself, the HIV itself, or other environmental factors.

## OBJECTIVE

This study aimed to perform a literature review to identify possible changes in the incidence of neoplasms in patients with HIV infection following subject to antiretroviral therapy.

## METHODOLOGY

**Figure 1:**
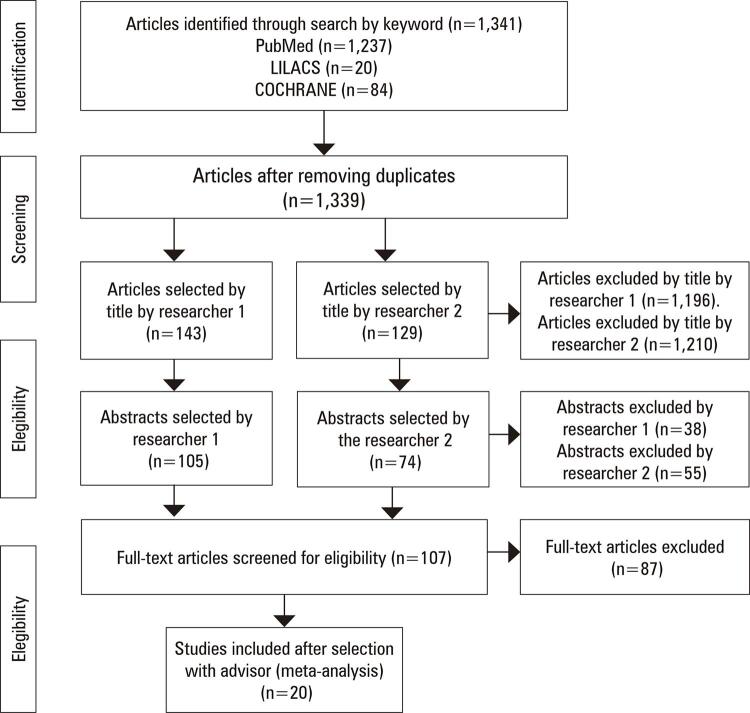
PRISMA flowchart of article selection

A horizontal review was performed based on the PRISMA statement.^(8)^ The review was carried out by two researchers independently on December 24 and 31, 2021. The following databases were searched: MEDLINE, LILACS and COCHRANE, using the following terms: “(cancer OR neoplasm) AND (hiv OR aids) AND (haart OR highly active antiretroviral therapy),” screening for publications dated from 2010 till now. After excluding duplicate articles, the researchers first screened the publications by title, followed by reading the abstracts independently in a standardized manner. Subsequently, the publications were selected after reading the full articles under the guidance of a senior researcher. The search and selection processes are illustrated in figure 1.

The inclusion criteria comprised articles extracted from the databases after screening them using the keywords as detailed above; published complete papers and abstracts, consistent with the theme “neoplasms in PLHIV after the antiretroviral therapy era”; in English; and those relevant to the objective of our literature review. A minimum follow-up time in each study was not defined.

The exclusion criteria comprised articles focusing on a specific neoplasm or on cancer treatment or survival rate, referring to patients with a diagnosis of cancer prior to HIV, with an emphasis on specific groups of the population, such as pregnant women, children, men who have sex with men (MSM), and people deprived of their liberty; articles that did not address neoplasia during the antiretroviral therapy era and/or those that did not include antiretroviral therapy in the discussion; studies with a small sample size (less than or equal to 100 patients); and animal studies.

The PRISMA flowchart in figure 1 illustrates the process of selection and exclusion of studies.

After finalizing the studies under the guidance of the senior advisor, the researchers developed a table that was filled out with the main information of the included studies that was based on the Cochrane Consumers and Communication Review Group’s data extraction template. Data were collecting including title, authors, citation, year of publication, number of patients, objective, study design, methodology, result, conclusion, and the researcher’s considerations. To avoid publication bias, the Zotero platform was used to juxtapose the information and exclude duplicate articles.

In view to reduce bias, the specific characteristics of each study were analyzed. The following topics were then analyzed and discussed: the declaration of risk of bias, possible conflicts of interest, generation of randomness, anonymity, and blinding. We then observed whether there were any common tendencies between the items evaluated in each article.

## RESULTS

Through the database search, 1,341 results were identified, two of which were duplicates. The two researchers selected 143 and 129 articles, respectively, based on the title. After reading the abstracts of each article, 105 and 74 articles were selected by the researchers 1 and 2. After analyzing and discussing these articles with a senior researcher, 20 articles were selected.

The main characteristics analyzed in each selected study are presented in table 1.^(9-28)^

**Table 1:** Individual characteristics of the selected studies

Reference	Population	Intervention	Control Group	Objective	Study design
Sachdeva et al.^(9)^	2,800 HIV-infected patients	Analysis of records		To determine the type and frequency of neoplasms in the population studied	Retrospective cohort
Franceschi et al.^(10)^	9,429 patients with HIV/AIDS	Analysis of nine records from Swiss cancer centers		To assess changes in cancer incidence in the pre-HAART, early HAART, and late HAART periods	Retrospective cohort
Reed et al.^(11)^	5,112 patients with HIV/AIDS and 3,578 patients with HIV/AIDS from a previous study.	Comparison of information between the current study and the study conducted by the research group ten years earlier		To compare data between the two studies in order to assess trends in neoplasms	Cohort/ retrospective cohort
Polesel et al.^(12)^	21,951 patients diagnosed with AIDS	Analysis of AIDS records and of Italian cancer data		To estimate changes in cancer incidence in individuals with HIV/AIDS in Italy before and after the introduction of HAART	Retrospective cohort
Spagnuolo et al.^(13)^	6,495 patients with HIV-infected	Data collection from patients infected with HIV between 1991 and 2010 at a single center		To describe survival in patients with HIV and diagnosis of AIDS-defining and non-AIDS-defining neoplasms	Retrospective cohort
Shiels et al.^(14)^	Number of 883,001 (estimated US population living with AIDS between 1991 and 2005)	Data analysis of AIDS prevalence and cancer rates		To assess trends in cancer burden among people with AIDS in the US during 1991-2005, which were distributed into pre-HAAT, HAART, and late HAART periods	Retrospective cohort
Simard et al.^(15)^	472,378 PLHIV/AIDS in 15 US states (1980 and 2008)	Link of AIDS records with cancer records.		To assess the impact of HAART on trends of mortality and cancer incidence in the population studied	Retrospective cohort
Achhra et al.^(16)^	Total of 4,111 patients (control group and group using interleukin-2)	Comparison between data (clinical evaluation, CD4 count and HIV RNA copy count) of patients included in the two groups	Patients not using interleukin-2 n=1,971	To explore the role of antiretroviral therapy and investigate the association between immunodeficiency, viremia and neoplasms	Retrospective cohort
Yanik et al.^(17)^	> 25 thousand PLHIV who started antiretroviral therapy between 1996 and 2011	Analysis of records from eight university centers		To assess trends in cancer incidence rates after initiation ofantiretroviral therapy.	Retrospective cohort
Franzetti et al.^(18)^	7,466 PLHIV	Analysis of patient data from a single center in Milan		To examine the incidence rates and characteristics of non-AIDS-defining neoplasms	Retrospective cohort
Cobucci et al.^(19)^	21 selected articles (N>600 thousand PLHIV/AIDS)	Systematic review using MOOSE guidelines		To assess the impact of HAART on the incidence of AIDS-defining and non-AIDS-defining neoplasms	Systematic review
Crum-Cianflone et al.^(20)^	2,238 PLHIV	Clinical interviews, physical exams, and collection of medical record data		To determine the effect of HIV control on cancer in extensive use of antiretroviral therapy.	Retrospective cohort
Castel et al.^(21)^	8,800 PLAIDS residents of Washington between 1996 and 2006	Analysis of Washington AIDS and cancer records during the study period		To assess the incidence and survival rates of cancer in PLAIDS during the early HAART and late HAART era	Retrospective cohort
Hernández-Ramírez et al.^(22)^	44,8258 PLHIV	Analysis of PLHIV records from nine US states and the link with cancer records		To describe the spectrum of cancer risk among HIV-infected people in the US during the HAART era	Retrospective cohort
Tanaka et al.^(23)^	87,109 PLAIDS in São Paulo between 1980 and 2013	Link between AIDS and cancer case records in the city of São Paulo	General population of São Paulo	To analyze trends in cancer incidence among residents of São Paulo with HIV compared to the general population	Retrospective cohort
Nizami et al.^(24)^	252 autopsies of HIV-infected patients	Analysis of autopsy records of HIV-infected patients		To analyze trends in the cause of death of HIV-infected patients in a New York hospital	Retrospective cohort
Chammartin et al.^(25)^	8,318 PLHIV between 2006 and 2016	Data collection on adverse events of antiretroviral therapy in PLHIV from Europe, Australia and United States of America		To estimate the difference in long-term risk for cancer with immediate antiretroviral therapy strategy	Retrospective cohort
Arora et al.^(26)^	1,258 PLHIV	Retrospective and prospective analysis of PLHIV from a medical center in India for seven years		To analyze the prevalence pattern and changing trends of neoplasms in PLHIV	Retrospective cohort
Neuhaus et al.^(27)^	5,472 PLHIV (SMART study) 4,111 PLHIV (ESPIRIT study)	Patients randomized to CD4-guided antiretroviral therapy or continuous use of antiretroviral therapy (SMART study) Patients randomized to interleukin-2 or continuous antiretroviral therapy (SPIRIT study)	Continuous use of HAART (SMART) Continuous use of HAART alone (SPIRIT)	To assess cumulative mortality six months after AIDS and non-AIDS-related events in two international studies	Retrospective cohort
Micheletti et al.^(28)^	261 necropsies of patients diagnosed with HIV	Analysis of necropsy information between 1989 and 2008		To evaluate an increase or decrease in the number of neoplasms after the introduction of HAART	Retrospective cohort

HIV: human immunodeficiency virus; AIDS: human acquired immunodeficiency syndrome; HAART: highly active antiretroviral therapy; PLHIV: people living with HIV; PLAIDS: people living with AIDS; SMART: strategies for management of anti-retroviral therapy; ESPIRIT: evaluation of subcutaneous proleukin in a randomized international trail; MOOSE: Meta-analysis Of Observational Studies in Epidemiology.

Among the studies selected for the systematic review, only one presented a systematic review^(19)^ and 19 were cohort studies. Most studies were retrospective observational studies and two were prospective articles.^(20,25)^

The studies included involved a total number of 2,605,869 patients with HIV/AIDS on antiretroviral therapy.

Among the 20 selected studies, ten were conducted in a single center^(9,11,13,16,18,21,23,24,26,28)^ and the other ten presented multicentric data collection.^(10,12,14,15,17,19,20,22,25,27)^ Regarding location, a prevalence was observed in the American and European continents, with ten articles from the United States, five from Europe, two from Brazil, one from India and two involving Europe-USA-Australia.

As a primary outcome, the studies assessed the incidence or risk of cancer in PLHIV/AIDS patients. In one study, the primary outcome was the survival of patients with HIV infection after a diagnosis of neoplasia.^(13)^

The results of all the articles selected in the review are described in table 2, considering the individual characteristics of each study (reference, year, and location). Moreover, relevant data to analyze this study’s hypotheses (reduction or increase in AIDS-defining and non-AIDS-defining neoplasms during the antiretroviral therapy era) are included.

**Table 2:** Findings of each selected study

Reference	Year	Location	Defining neoplasm	Non-defining neoplasm
Sachdeva et al.^(9)^	2016	USA	Decrease	Decrease
Franceschi et al.^(10)^	2010	Switzerland	Decrease	No change
Reed et al.^(11)^	2010	USA	Decrease	Increase
Polesel et al.^(12)^	2010	Italy	Decrease	Increase (gross number)
Spagnuolo et al.^(13)^	2012	Italy	Decrease	Increase
Shiels et al.^(14)^	2011	USA	Decrease	Increase
Simard et al.^(15)^	2011	USA	Decrease	Increase
Achhra et al.^(16)^	2010	Amsterdam	Decrease	Increase
Yanik et al.^(17)^	2013	USA	Decrease	Increase
Franzetti et al.^(18)^	2013	Italy		Increase
Cobucci et al.^(19)^	2015	Italy, United Kingdom, USA, Switzerland, Australia, France, Europe	Decrease	Increase
Crum-Cianflone et al.^(20)^	2015	USA	Decrease	Increase
Castel et al.^(21)^	2015	USA	Decrease	Decrease
Hernández-Ramírez et al.^(22)^	2017	USA	Decrease	Decrease
Tanaka et al.^(23)^	2017	Brazil	Decrease	Decrease
Nizami et.^(24)^	2020	USA	No change	No change
Chammartin et al.^(25)^	2021	Europe, Australia and USA	No significant change	No significant change
Arora et al.^(26)^	2021	India	Decrease	Increase
Neuhaus et al.^(27)^	2010	Denmark-England-Australia (SMART) and Multicentric with 23 countries (North and South America, Europe, Australia and Asia) (ESPIRIT)	Higher frequency and higher morbidity compared to non-defining	Lower frequency and lower morbidity
Micheletti et al.^(28)^	2011	Brazil	No change	No change

USA: United States of America; SMART: strategies for the management of anti-retroviral therapy; ESPIRIT: evaluation of subcutaneous proleukins in a randomized international trial.

Among the 20 selected studies, 19 established a comparison between neoplasms in the period before the use of antiretroviral therapy and the period after the use of these medications, of which one did not address AIDS-defining neoplasms. Fifteen studies revealed a reduction in the overall incidence of AIDS-defining neoplasms during the antiretroviral therapy era, and three studies indicated no change. Regarding non-AIDS-defining neoplasms, the results are more controversial; 12 studies indicated an increase in overall incidence, four indicated no change, and three indicated reductions. One of the articles reported a different comparison from the others, indicating a higher prevalence of non-AIDS-defining neoplasms than AIDS-defining neoplasms, both in the post-HAART period. In addition, regarding the subtypes of neoplasms, most articles have highlighted a reduction in the incidence of KS and NHL, in addition to an increase in the prevalence of some non-AIDS-defining neoplasms (HL, anal cancer, lung cancer, and liver cancer), especially those related to risk behaviors and oncogenic viral infections.

Among the results presented, we highlight the specific increase of some cancer subtypes during the antiretroviral therapy era in PLHIV/AIDS compared with those in the pre-antiretroviral therapy period, such as HL;^(9,10,14,15,19)^anal cancer;^(9,10,14,15,17-19,21,23)^ liver cancer;^(10,12,14,15,19)^ lung cancer;^(12,14,15,19,23)^non-melanomatous skin cancer;^(10)^prostate cancer;^(14,19)^ breast cancer;^(18)^ vulvar cancer;^(18)^ and oral cancer.^(21)^ The possible reasons of why this occurs are discussed later.

Another interesting point is that 17 studies were developed along the USA-Europe-Australia axis; the remaining two were Brazilian, and one was from India.

**Figure 2:**
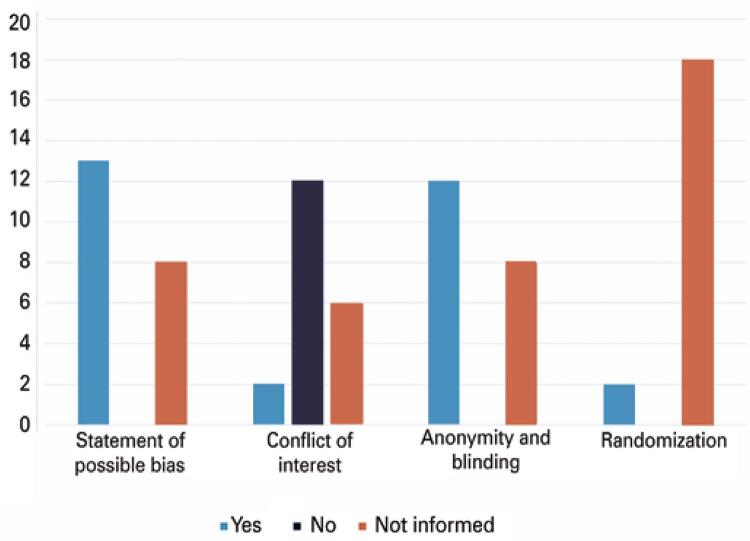
Assessment of risk of bias

We assessed the risk of bias in each study and carried out quantitative analyses of the main characteristics, indicating greater or lesser propensity to bias (Figure 2).

Considering the analysis of the information depicted in figure 2, eight studies did not present information on blinding. However, 19 of the studies were observational studies.

Thirteen selected articles self-declared a possible risk of bias as one of their limitations, mainly owing to the lack of specific information in data collection. In addition, in 19 of the 20 studies, a database was used to collect patient data (Table 1).

## DISCUSSION

Why has there been an increase in the incidence of non-AIDS-defining neoplasms in the antiretroviral therapy era?

The incidence of cancer in PLHIV has changed with the advent of antiretroviral therapy; cases of AIDS-defining neoplasms have decreased, whereas those of non-AIDS-defining neoplasms have increased. Although the decrease in the incidence of AIDS-defining neoplasms can be attributed to the improved immunity resulting from HAART, the reason for the increase in non-AIDS-defining neoplasms has not yet been fully explained. Some reasons considered for this include greater survival of PLHIV, risk factors present in this population, improved diagnostic tests, oncogenic effects of antiretroviral therapy, chronic immune activation and inflammation, and the oncogenic activity of HIV itself.^(29-32)^

The main factor to be considered is that antiretroviral therapy has contributed to the immunological restoration of HIV-infected patients, aiming at an undetectable viral load and resulting in increased survival in this population. Therefore, two issues are noteworthy: as the number of deaths resulting from immunosuppression decreases, the risk of death from other causes decreases.; the aging population of PLHIV, who are more susceptible to chronic diseases, including cancer, than the normal population.^(13,33)^ However, the incidences of some types of cancer in this population is higher than those in the general population, and occurs at a younger age, requiring further investigation.^(32)^

Studies on the incidence of cancer in PLHIV treated with different antiretroviral drugs have shown differences in their roles. In some studies^(33,34)^ it was found that PIs can have both antiviral and anti-angiogenic effects, acting on different tissues and possibly reducing the risk of KS and NHL.^(35)^ However, some studies have related the use of antiretroviral therapy to a lower incidence of non-AIDS-defining neoplasms.^(36)^ Meanwhile, drugs such as zidovudine and zalcitabine have been associated with carcinogenesis in experimental animal studies,^(37)^ and drugs from the non-nucleoside reverse transcriptase inhibitor class have been linked to increased HL.^(38)^ Further research is needed to determine the effect of antiretroviral therapy on the risk of PLHIV developing infections.

Although immunosuppression is linked to a higher risk of cancer development, the association between HIV infection and an increase in non-AIDS-defining neoplasms in the antiretroviral therapy era is still being examined. The hypotheses are an indirect relationship, given the long-term exposure to immune suppression, or a direct relationship, with the possibility that HIV itself is oncogenic.^(39)^ The possible oncogenic role of HIV is attributed to the fact that the virus causes genetic alterations through the activation of proto-oncogenes and inhibition of tumor suppressor genes.^(32,40)^ Although the role of HIV in this hypothesis is inconclusive, it is known that PLHIV presents higher rates of risk behaviors directly related to the development of neoplasms.^(41)^

### Comparison between cancer rates in PLHIV and in the general population

Although the aging PLHIV population is an important risk factor for the development of neoplasms, the increase in the incidence of non-AIDS-defining neoplasms cannot be explained solely by this fact. This is shown as in cohorts with individuals of the same age whereby the risk of neoplasms was higher in HIV-infected individuals than in the general population.^(42)^

In a cohort study of PLHIV in British Columbia,^(42)^ the incidence of non-AIDS-defining neoplasms was compared with the expected incidence in the general population. The result was an overall standard incidence rate of 2.05. Males aged 20–39 years had a standard incidence rate of 5.45. The incidence rate was higher with individuals with lung^(3,42)^ and anal cancers.^(7,43)^

In a study conducted in France, the age at which neoplasia in PLHIV was identified was compared to that in the general population. Significantly younger age at diagnosis was identified in HIV-infected individuals with lung cancer (3.3 years), HL (1 year), and liver cancer (10.1 years). In addition, the age-standardized incidence rates for the four types of cancer were higher among HIV-infected men and women than in the general population during antiretroviral therapy periods.^(30)^

Thus, when comparing the general population with people living with AIDS (PLAIDS), HIV-infected individuals have a higher risk of developing specific subtypes of neoplasms. However, studies on the factors leading to this increased precocity in the occurrence of cancer is required.

In addition to differences in incidence, differences in survival can also be assessed. Although antiretroviral therapy increases the survival of HIV-infected patients with neoplasms, survival for AIDS-defining and non-AIDS-defining neoplasms is still lower than that of the rest of the population.^(13,43,44)^ This may be due to factors such as an advanced stage of cancer at the time of diagnosis, a more aggressive nature of the cancer, and a higher incidence of concomitant comorbidities such as viral hepatitis.^(13)^

With an increase in non-AIDS-related neoplasms in the antiretroviral therapy era, it is necessary to understand the risk factors for cancers involving PLHIV in the context of improved immunity. Some factors must be considered, such as age, sex, transmission route, alcohol consumption and smoking, duration of HIV infection and treatment, and the presence of previous diagnoses of AIDS and other neoplasms.^(31)^

The main risk factors for AIDS-defining neoplasms are the absence of antiretroviral therapy, reduced CD4^+^T lymphocyte count, detectable HIV viral load, and previous AIDS diagnosis. In contrast, for non-AIDS-defining neoplasms, advanced age, previous AIDS diagnosis, and low CD4+T cell counts can be highlighted as risk factors.^(45)^

A direct relationship was found between the duration of immunosuppression and the risk of neoplasms; in each year that the HIV-infected patient had a T cell count below 200 cells/mm^3^, the risk of developing cancer increased by 36%.^(46)^ The higher risk is particularly noticeable in neoplasms related to infections, including oncogenic viruses, such as HHV-8 and hepatitis B and C.^(39,45)^ Meanwhile, advanced age was shown to be a risk factor for all cancers, except KS, and non-AIDS-defining neoplasms that were not lymphomas or HPV-related were associated with a higher risk.^(17)^

The higher risk of neoplasms in PLHIV in the antiretroviral therapy era is usually associated with oncogenic viral coinfections and risky behaviors in this population, such as smoking, drug use, and alcohol abuse.^(18,46)^ Regarding infections such as HPV, HVC, HVB, and EBV, immunodeficiency is associated with higher persistence, reactivation, and progression.

The incidence of HPV infection is higher in PLHIV, which has a proven association with anogenital cancer.^(18)^ Thus, it is not unexpected that compared to the general population, a 30-fold higher incidence rate was shown in a Swiss cohort^(10)^ and that it affects more MSM around 45–50 years of age.^(47)^ In the post-antiretroviral therapy era, a four-fold higher risk of developing anal cancer was found, that was probably related to the increase in screening and consequent diagnosis of this type of cancer.^(19)^

There is also a higher risk of liver cancer among HIV-infected people because, there is often a higher prevalence of hepatitis B and C, and co-infection with these viruses appears to increase the risk of developing cirrhosis, end-stage renal disease, and hepatocellular carcinoma.^(19)^

In the antiretroviral therapy era, there has been an increase in the incidence of lung cancer, with smoking being the main risk factor.^(19)^ Approximately 35–70% of PLHIV are smokers, while in the general population, the rate is 20%.^(48,49)^ Given this high prevalence, there is also a high incidence of cancer in the oral cavity and larynx.^(50)^

## LIMITATIONS

This literature review, based on the PRISMA method, has some limitations, such as the lack of homogenization of the selected articles. In addition, selection bias could have been present because the systematic review selected only articles that had already been published; therefore, there was a higher probability of presenting statistically relevant results to the detriment of studies that were not published due to less relevant data.

Finally, the review process was limited because of the restriction to include publications in English only.

## CONCLUSION

With the selection of 20 studies in our systematic review, we concluded that there was an overall reduction in the incidence of AIDS-defining neoplasms during the antiretroviral therapy era compared to the pre-antiretroviral therapy era. Furthermore, 12 articles reported an overall increase in the incidence of non-AIDS-defining neoplasms after antiretroviral therapy. New questions arise in this scenario, such as the reason for the increase in the incidence of these neoplasms, whether there is a difference in the incidence in people living with HIV compared to the general population, and even the present risk factors linked to this event.

The hypotheses addressed to understand this higher incidence reinforce the speculation of a multifactorial explanation, comprising greater survival of the HIV-infected population and greater vulnerability to diseases resulting from aging, such as neoplasms. However, because a higher risk of non-AIDS-defining neoplasms was observed in people living with HIV than in the general population occurring at a younger age, other justifications should also be considered, such as the possible influence of the drugs used in antiretroviral therapy, greater accuracy of diagnostic and screening tests, and/or immune surveillance capacity impaired by immune activation and chronic inflammation induced by HIV.

It is also vital to investigate the risk factors involved in these situations, such as advanced age, previous diagnosis of AIDS, low CD4+T cell count, viral co-infections, and risk behaviors common to people living with HIV, such as smoking and alcohol consumption. This, together with the compromised control of viral replication, contributes to a higher incidence of neoplasms related to viral infections and a lower survival rate compared with the general population.

Briefly, since the era of antiretroviral therapy, there has been a noticeable change in the survival and epidemiological profiles of people living with HIV and neoplasia, raising new questions and challenges . Although this study provides a holistic view on the matter and discusses important points about this new era, further studies focusing on the oncogenic role of HIV and antiretroviral therapy, and on specific subtypes of neoplasms in people living with HIV in comparison with the general population are needed. In addition, the relevance and urgency of screening for neoplasms in HIV-infected populations should be emphasized. As previously discussed, people living with HIV are more susceptible to neoplasms. Therefore, prevention and screening protocols with greater attention to this population are required.
